# In-situ X-ray monitoring of solidification and related processes of metal alloys

**DOI:** 10.1038/s41526-023-00321-z

**Published:** 2023-09-06

**Authors:** G. Reinhart, D. J. Browne, F. Kargl, F. García-Moreno, M. Becker, E. Sondermann, K. Binder, J. S. Mullen, G. Zimmermann, R. H. Mathiesen, W. H. Sillekens, H. Nguyen-Thi

**Affiliations:** 1 Aix-Marseille Univ, Université de Toulon, CNRS, IM2NP UMR 7334, 13397 Marseille, France; 2 School of Mechanical and Materials Engineering, University College Dublin, Belfield 4, Dublin, Ireland; 3 Institut für Materialphysik im Weltraum, Deutsches Zentrum für Luft- und Raumfahrt (DLR), 51170 Köln, Germany; 4 Institute of Applied Materials, Helmholtz-Zentrum Berlin für Materialien und Energie, Hahn-Meitner-Platz 1, 14109 Berlin, Germany; 5 Access e.V., Intzestraße 5, 52072 Aachen, Germany; 6 Department of Physics, Norwegian University of Science and Technology (NTNU), N-7491 Trondheim, Norway; 7 European Space Agency – ESTEC, Keplerlaan 1 Postbus 299, 2200 AG Noordwijk, The Netherlands

**Keywords:** Materials science, Structural materials

## Abstract

X-ray radioscopy enables the in-situ monitoring of metal alloy processes and then gives access to crucial information on the dynamics of the underlying phenomena. In the last decade, the utilisation of this powerful imaging technique has been adapted to microgravity platforms such as sounding rockets and parabolic flights. The combination of microgravity experimentation with X-ray radioscopy has resulted in a leap in the understanding of fundamental science and has opened new paths in the fields of materials science. The present review focuses on the short history of this research, which includes facility developments, microgravity experiments and results obtained by partners of the XRMON (In-situ X-Ray MONitoring of advanced metallurgical processes under microgravity and terrestrial conditions) research project in the framework of the MAP (Microgravity Application Promotion) programme of the European Space Agency. Three illustrative research topics that were advanced significantly through the use of X-ray radioscopy will be detailed: solidification of metal alloys, metallic foam formation and diffusion in melts.

## Introduction

The accurate control of metallurgical processes requires a clear understanding of complex mechanisms that act at different scales in time and space, such as grain nucleation, diffusion of chemical species, dendritic growth, fluid flow, development of gas bubbles etc.^1^. The study of metallurgical processes is made even more difficult on Earth where these mechanisms are combined with gravity-related phenomena such as buoyancy-driven convection and sedimentation. These are a major source of disturbing effects, significantly modifying, or overshadowing other physical phenomena^2–4^. Nevertheless, if pure diffusive conditions can be established to collect benchmark data^5^, the impact of gravity can be highlighted by comparison with ground experiments. Furthermore, in-situ and real-time observation must be retained as a method of choice to improve the understanding of the underlying phenomena because most of them are dynamic^5^. Recent developments of more powerful microfocus laboratory X-ray sources along with modern X-ray detectors have paved the way for the application of X-ray imaging to microgravity experiments. Among the various X-ray imaging techniques that can be implemented to carry out in-situ and time-resolved observation^6^, X-ray radioscopy is the most used as it is the simplest to achieve. This technique consists principally of illuminating an investigated sample with an X-ray beam and collecting the transmitted beam on an X-ray sensitive camera to record in a time-resolved manner a two-dimensional image corresponding to the projection of the crossed objects.

The adaptation of X-ray radioscopy systems into experimental devices used aboard microgravity platforms has been the task of XRMON (In-situ X-Ray MONitoring of advanced metallurgical processes under microgravity and terrestrial conditions) research project partners in the framework of the MAP (Microgravity Application Promotion) programme of ESA (European Space Agency). The XRMON project emerged as a continuation of an academic Topical Team network that started in 2004, in parallel to feasibility studies on hardware commissioned by ESA^7^.

It started in 2006 and ended in 2018 after two renewals, gathering both academic and industrial partners^8^. The XRMON partners have been involved in the development of laboratory experimental devices that can record radiographs with both sufficient contrast and low signal-to-noise ratio, allowing observations to be made with temporal and spatial resolutions that are appropriate to study the dynamics of the investigated phenomena. In addition, the devices have been designed in terms of weight, space constraint and energy consumption to be operable on microgravity platforms such as sounding rockets and parabolic flights. Hitherto, several sounding rocket missions and parabolic flight campaigns have been successfully completed and are listed in Table 1.

**Table 1:** List of sounding rocket missions and parabolic flight campaigns.

Research topics	Sounding rockets	Parabolic flights
Metallic foam formation	MASER-11 (2008)	ESA PF-46 (2007)
		ESA PF-51 (2009)
		ESA PF-65 (2016)
		ESA PF-67 (2017)
Diffusion in metallic melts	MAXUS-8 (2010)	
	MAPHEUS-4 (2013)	DLR PF-22 (2013)
	MAPHEUS-5 (2015)	DLR PF-24 (2014)
	MAXUS-9 (2017)	
Solidification of metal alloys		ESA PF-58 (2013)
	MASER-12 (2012)	DLR PF-22 (2013)
	MASER-13 (2015)	DLR PF-24 (2014)
	MAPHEUS-6 (2017)	ESA PF-60 (2014)
	MAPHEUS-7 (2018)	ESA PF-61 (2014)
	MASER-14 (2019)	ESA PF-64 (2016)
		DLR PF-36 (2021)

List of sounding rocket missions and parabolic flight campaigns where XRMON partners have carried out in-situ observations by using X-ray radioscopy.

The aim of the present paper is to provide an up-to-date overview of investigations carried out on microgravity platforms and devoted to the study of solidification of metal alloys, metallic foam formation, and diffusion in metallic melts by using X-ray radioscopy. A selection of scientific results obtained on the three topics illustrating the great potential of in-situ observation on Earth and particularly in microgravity conditions will be presented. Then, prospects for future developments and experiments in line with the SciSpacE Materials Science white papers^9^ will be discussed.

## Solidification of metal alloys

The solidification of metal alloys in a temperature gradient typically results in the formation of elongated columnar crystals, whereas a uniform temperature favours the formation of isotropic equiaxed grains. A change from columnar to equiaxed grain structure, commonly called columnar-to-equiaxed transition (CET), is often observed during industrial processes such as ingot casting^10^ or additive manufacturing^11^. On Earth, gravity-related phenomena such as buoyancy-driven convection and sedimentation are a major source of disturbing effects, making it difficult to investigate in detail the phenomena occurring during the grain structure formation^8^. However, the impact of gravity can be best highlighted by comparing the results of ground-based experiments to benchmark data collected in pure diffusive conditions which use microgravity platforms equipped with in-situ observation devices.

## Directional solidification

A facility named XRMON-GF (GF for gradient furnace) was developed for the study of directional solidification of aluminium-based alloys with X-ray radioscopy on board microgravity platforms. The facility consists of a Bridgman furnace allowing directional solidification to be performed with temperature gradients within the range 1–10 K/mm, coupled with an X-ray radioscopy system with an effective pixel size after magnification of 4 µm × 4 µm and a frame rate of 3 frames per second, which is satisfactory for studying the evolution of the solidification microstructure. The solidification of the sample (5 mm in width, 50 mm in length and 150 µm to 200 µm in thickness) is triggered by decreasing the heater temperature. Full details about the XRMON-GF module are available elsewhere^12,13^. XRMON-GF was successfully used during the MASER-12 and MASER-14 sounding rocket campaigns^14,15^. For all experiments, suitable timelines were defined to analyse in diffusive conditions the impact of gravity on (i) the columnar growth of non-refined Al-20wt.%Cu and (ii) the Columnar-to-Equiaxed Transition in grain-refined Al-20wt.%Cu. Ground reference tests were performed with the same experimental device and temperature profile, but for different sample orientations with respect to the gravity vector.

**Fig. 1:**
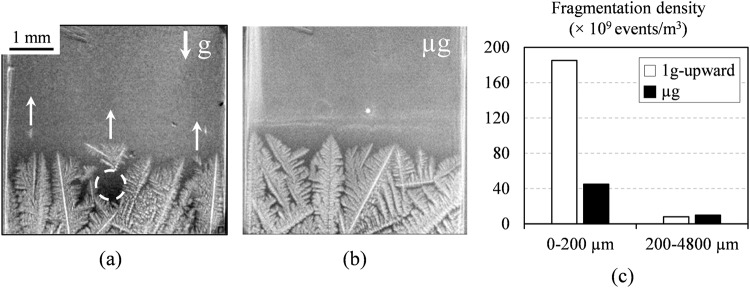
Investigation of columnar growth and fragmentation in Al-20wt.%Cu. Radiographs showing the columnar growth of an Al-20wt.%Cu alloy (**a**) with the sample in vertical position and (**b**) in microgravity^14^. **c** Fragmentation density in the top (0–200 µm from the solidification front) and bottom (200–4800 µm from the solidification from) of the mushy zone.

**Fig. 2:**
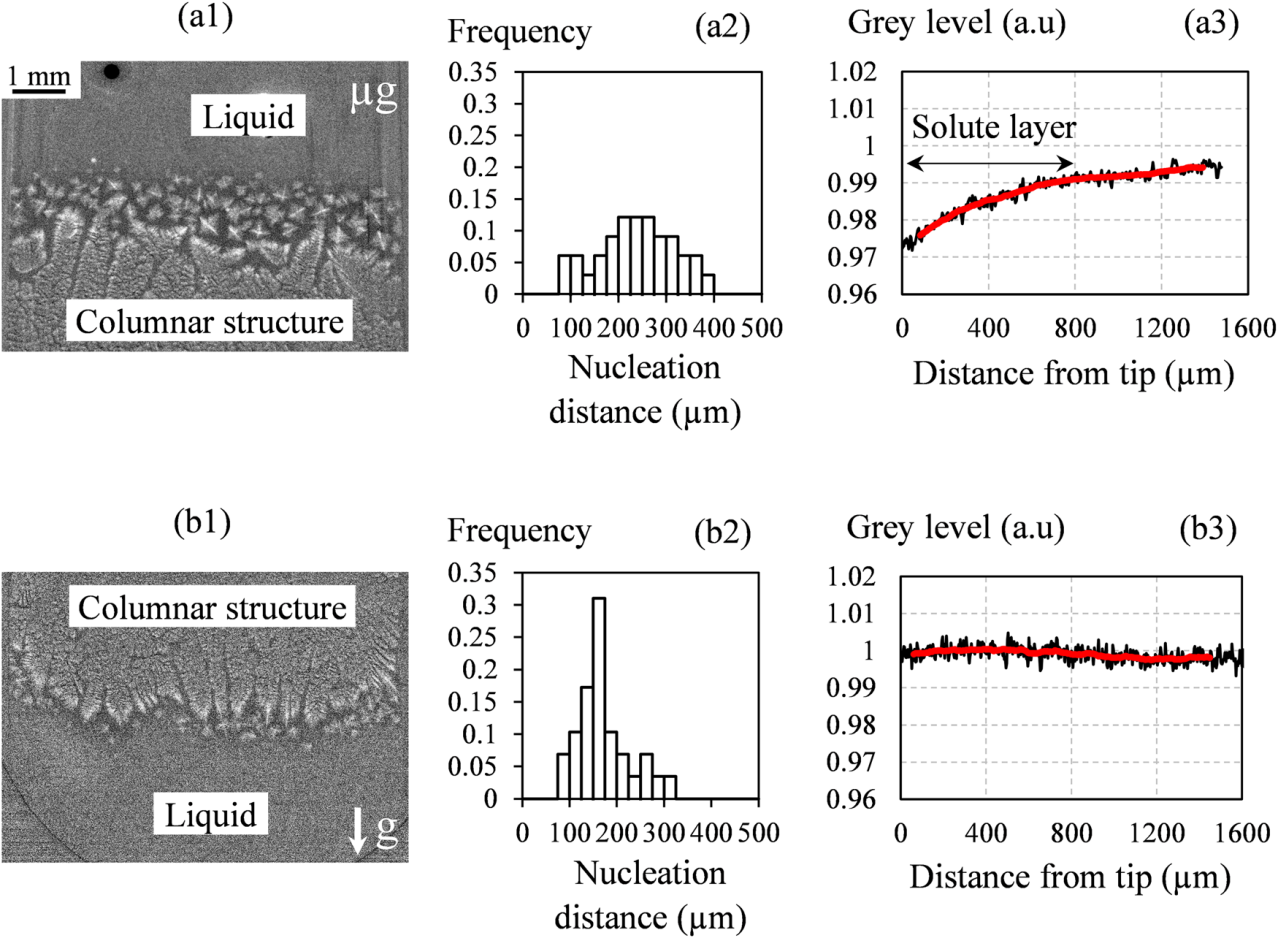
Study of the nucleation distance in refined Al-20wt.%Cu. Processed images showing the nucleation of equiaxed grains ahead of the columnar solidification front (**a**1) in microgravity condition, (**b**1) for the downward growth terrestrial configuration. Measured grain nucleation distance (**a**2) in microgravity condition, (**b**2) for the downward growth. Grey level variation ahead of the leading dendrite tip (**a**3) in microgravity condition and (**b**3) in the downward growth configuration^15^.

Figure 1 displays two images showing the development of a columnar microstructure in a non-refined Al-20wt.%Cu alloy for two experimental configurations^14^: in vertical position with the growth direction opposite to the gravity vector (upward configuration, Fig. 1a) and in microgravity (Fig. 1b). The growth of columnar dendritic grains is clearly visible in the radiographs. The impact of gravity is significant for the upward configuration. Solid fragments formed preferentially at the top of the mushy zone (Fig. 1c) and floated toward the hot zone because, for this concentrated alloy, the density of the solid is lower than the density of the surrounding liquid. Most fragments gradually melted in the hot zone, which could not have been deduced from post-mortem analyses. Some fragments got stuck in the thin crucible after floating for a short distance, leaving behind a liquid area (dashed white circle in Fig. 2a). Solid continued to grow slowly toward these liquid areas, rejecting solute and leading to the formation of segregated zones that eventually solidified after reaching the eutectic composition. No grain flotation was observed for the experiments in microgravity, due to the absence of buoyancy. A few grains nucleated on the sample oxide layer and were eventually incorporated to the columnar solidification front. Dendrite fragmentation was also observed but in smaller number (Fig. 1c) and deep in the mushy zone. Unexpectedly, the fragments moved toward the cold zone and this motion is attributed to liquid flow induced by solidification shrinkage that carried away the fragments^16^. The latter observations clearly highlight the interest of performing comparative studies between experiment on ground and in microgravity to evidence phenomena that would be otherwise overlooked.

The CET was then investigated in a refined Al-20wt.%Cu alloy. Grain refiners are often added to liquid alloy melts to increase volumetric nucleation density and encourage growth of small equiaxed grains^17^. Experiments were performed with an applied temperature gradient *G*_*app*_ = 10 K/mm and a slow cooling rate *R*_*1*_ = 0.08 °C/s to ensure a columnar solidification, followed by a rapid cooling rate *R*_*2*_ = 1 °C/s to activate the refining particles and, in that way, trigger the CET^15^. Figure 2 shows the new grains that nucleated ahead of the solidification front after the application of the rapid cooling rate for two experimental configurations: in microgravity (Fig. 2a1) and in vertical position, on Earth, with the growth in the same direction as the gravity vector (downward configuration, Fig. 2b1). In-situ observations allowed the nucleation distance to be measured for the first layer of grains (Fig. 2a2, b2) and the measurements show that the grains nucleated significantly farther from the columnar front in microgravity. This difference is attributed to a modification of the solute profile ahead of the solidification front by convective flow on Earth. This was confirmed by the direct measurement of the grey-level profile above columnar dendrites (Fig. 2a3, b3), which showed that the solute layer, and therefore the extent of the undercooled region where new grains can nucleate, is larger in microgravity, but nearly wiped out by convection in the case of downward growth on ground.

## Equiaxed solidification

**Fig. 3:**
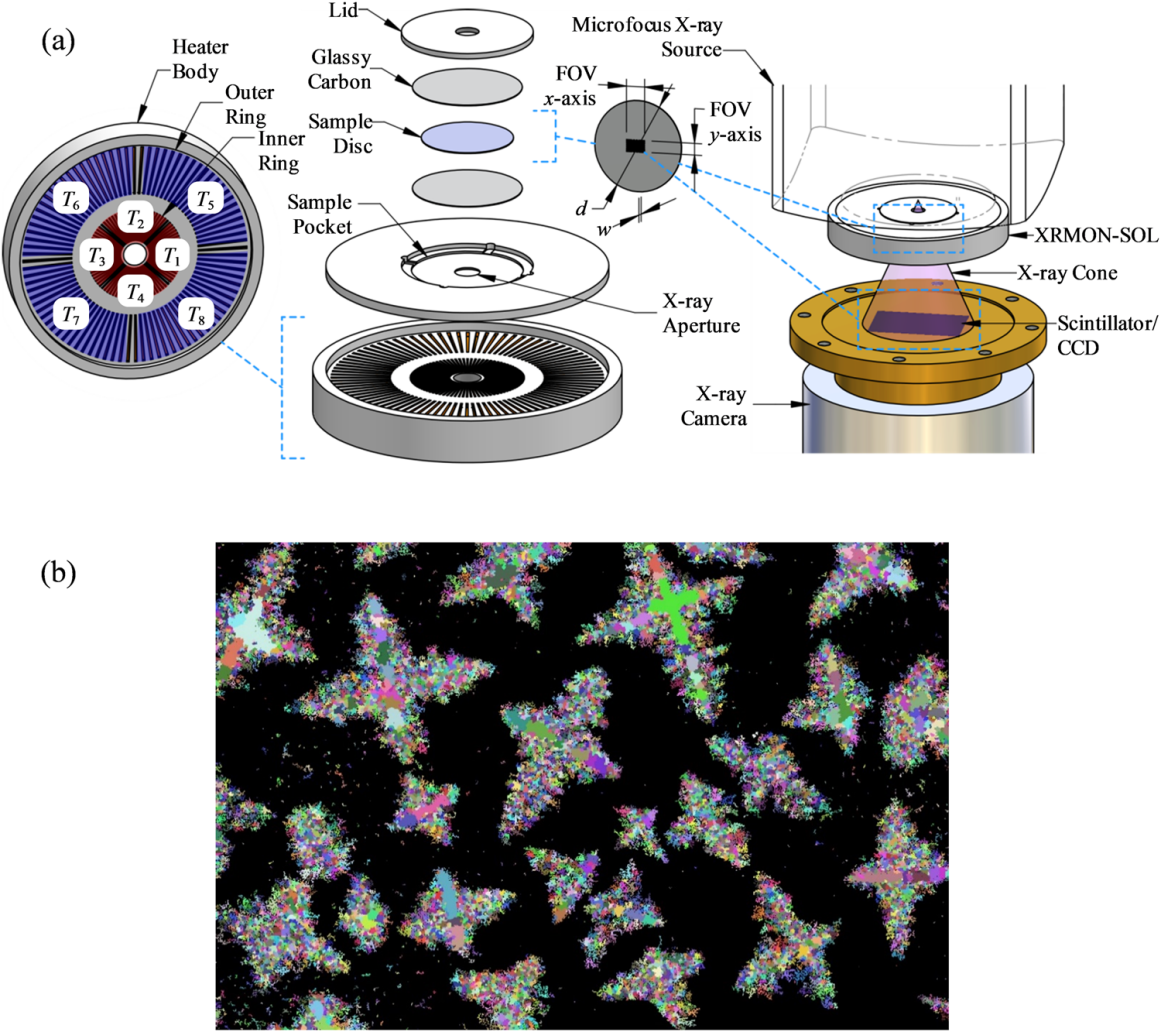
XRMON-SOL device and analysis of equiaxed growth from MASER-13. **a** Schematic illustration of XRMON-SOL construction and integration with in-situ X-ray diagnostics. Labels T_1→8_ indicate the relative location and arrangement of eight independently regulated heater coils. Dimensions d and w denote the sample diameter (21 mm) and thickness (0.2 mm), respectively. FoV x-axis and y-axis represent the physical extent of the X-ray field of view relative to the sample diameter, horizontally (~4.1 mm) and vertically (~2.7 mm), respectively. **b** dendrites from the MASER-13 sequence as automatically identified and separated by Machine Learning; individual colours represent sub-dendrite areas^22^ which are added computationally as the dendrite grows, are associated to one particular dendrite, and thus ensure that each dendrite retains its own identity up to and beyond impingement with other dendrites (the so-called coherency point) and the final eutectic solidification.

The target macro-structure for most as-solidified engineering components consists of fine equiaxed grains, as this leads to higher strength and isotropic properties. Equiaxed solidification^18^ occurs at very low values of thermal gradient (*G*), but XRMON-GF was designed to operate at non-zero *G*. As a result, we designed and developed a furnace particularly suited to achievement, and in-situ observation, of nucleation and growth of equiaxed crystals in microgravity. The furnace, called XRMON-SOL, was circular in shape^19^, and encapsulated a thin disc-shaped sample of an Al-Cu alloy (Fig. 3a). In initial trials on Earth, nucleation occurred at random locations within the 4.1 mm × 2.7 mm field of view (FoV), indicating a locally spatially isothermal sample, and growth was uniform equiaxed. This provided benchmark data used for computational modelling of equiaxed solidification^20^. The XRMON-SOL furnace was installed on the MASER-13 sounding rocket, which was prepared for launch. The microgravity flight took place on 1 December 2015, and was an operational and scientific success – providing the first ever in-situ X-ray video of a complete sequence of polycrystalline equiaxed solidification of a metallic alloy in space^21^. The grain-refined Al-20wt.% Cu alloy was completely melted and re-solidified during the 6-minute microgravity period. Nucleation and growth occurred uniformly across the FoV, but the grains themselves were motionless (i.e. they did not translate or rotate) during the growth period until after impingement and just before the final eutectic arrest, where some grain rotation occurred due to volumetric shrinkage^8^, which is not a gravitational effect. The output provides unique experimental data for validation of models of microstructural evolution of diffusion-controlled equiaxed alloy solidification. Machine Learning (ML) techniques are currently being used to automate the analysis and quantification of the X-ray sequences^22^. Figure 3b shows an example of the ML-classification of dendrites (using sub-sets automatically identified as belonging to certain crystals) during solidification.

Additional near-isothermal solidification experiments have been carried out on two sounding rocket missions MAPHEUS-6 and 7 in the years 2016 and 2018, respectively, also using X-ray observation technology and a different isothermal furnace design^23^. Two Al-15 wt.% Cu samples in two individual solidification furnaces sharing one X-ray source have been solidified in microgravity during MAPHEUS-7, and one Al-46wt.% Ge sample during MAPHEUS-6^24^. Nucleation dynamics as well as dendrite and melt concentration evolution were monitored in-situ.

**Fig. 4:**
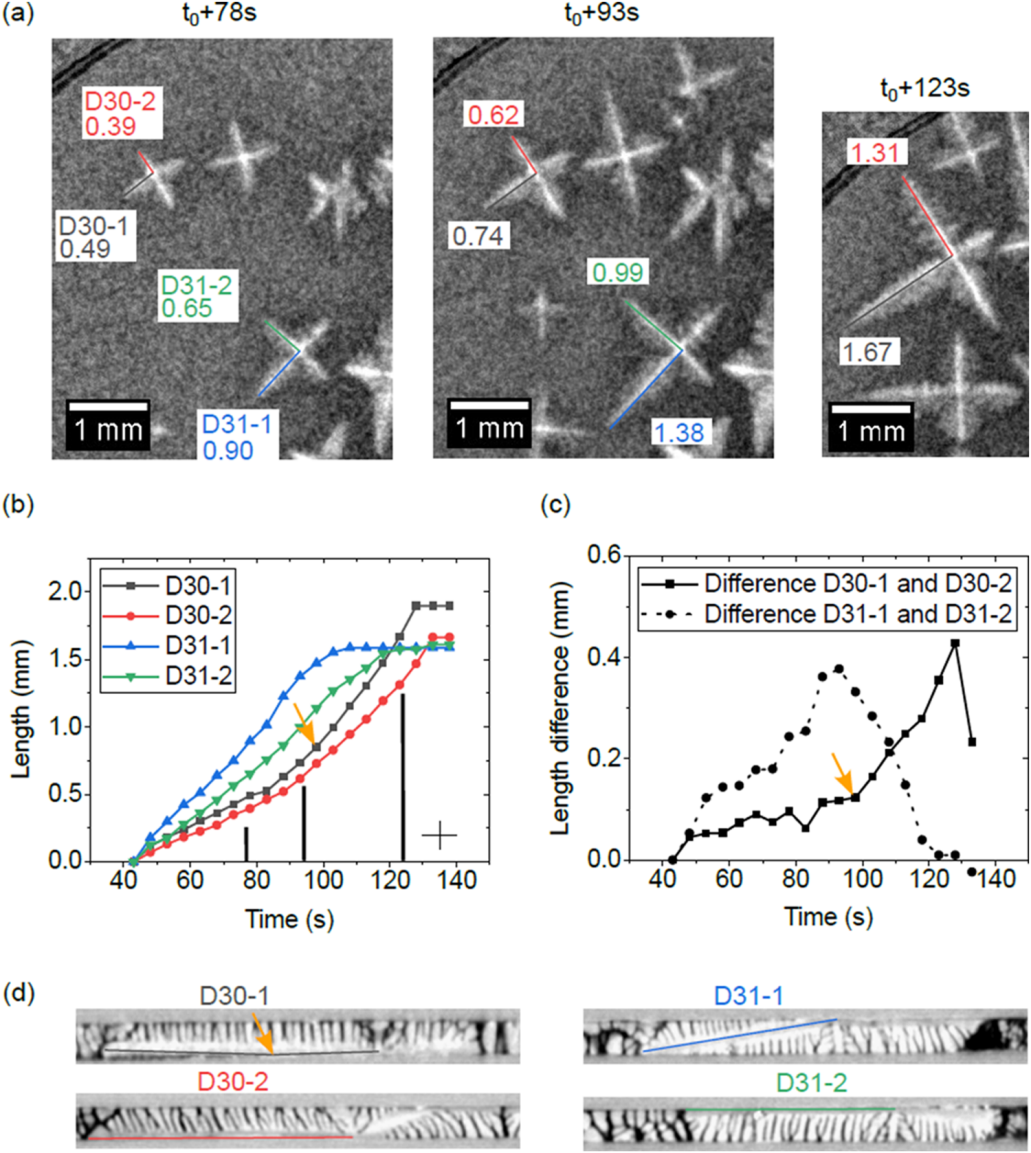
Analysis of equiaxed growth from MAPHEUS-7-2. **a** Radiographs of the microgravity equiaxed growth experiment (Al-15wt.% Cu sample). The lengths of selected dendrite arms are shown in millimetres. t_0_ corresponds to the time of the first dendrite nucleation in the sample. The black lines at the top left of the images are from the image correction process and are due to the projection of the edge of the furnace holding frame. **b** Dendrite arm lengths measured over time for the four dendrite arms D30-1, D30-2, D31-1 and D31-2. **c** The difference between the length measurement of D30-1 and D30-2 and of D31-1 and D31-2. **d** Post-flight X-ray tomography cross sections of the selected dendrite arms. The sections reveal the arm growth orientations of the dendrites, either along the sample boundary or diagonally into the sample. The orange arrow marks the point at which dendrite arm D30-1 detaches from the sample surface, resulting in an increase in growth rate.

To learn more about the orientation of the dendrites with respect to the sample geometry and about the interactions of the dendrite arms with the container walls, post-flight X-ray tomography and electron backscatter diffraction (EBSD) were performed on one Al-15wt.% Cu sample (MAPHEUS-7-2)^25^. This microgravity equiaxed growth experiment is used here to analyse the relationship between dendrite-boundary interaction and tip growth rates. Figure 4a shows three radiographs at different experiment times. Growth length measurements were performed on four dendrite arms of two dendrites that have started to nucleate temporally close together (D30 and D31). In Fig. 4b, the measured dendrite arm lengths are plotted against time, where t_0_ marks the onset of solidification of the sample. Overall, dendrite D31 grows faster than D30. This is due to a larger undercooling in the region of D31 during nucleation. The arms of the respective dendrites also show growth rate differences: Fig. 4c shows that the growth rate difference between D30-1 and D30-2 increases considerably from time t_0_ + 98 s onwards (orange arrow). Compared to dendrite D30, the growth rate difference between the dendrite arms D31-1 and D31-2 increases even faster. Looking now at the tomography sections along the dendrite trunks in Fig. 4d, we see that the growth rates always increase when the dendrite tips are not in contact with the sample surface but grow freely. This occurs whenever the dendrite orientations deviate from the in-plane <100> growth direction, which is the case for almost all dendrites in the sample, as confirmed by EBSD measurements^25^. The sample surface hinders the spatial growth of the dendrite tip, so that the preferred <100> growth direction cannot be maintained, with the consequence that the growth rate decreases. The observation made in mesoscopic simulations by Olmedilla et al. ^20^. that dendrite tips grow faster along the sample boundary could not be confirmed. However, because the simulations do not account for the kinking of the arms at the boundary and the experiments do not show dendrites with an ideal <100> growth orientation along the boundary, an accurate comparison is difficult. It remains to be investigated how the wetting behaviour depends on the alloy material and the boundary conditions of the sample environment.

## Metallic foam formation

The industrial production volume of metal foams is still low due to high manufacturing costs and insufficient material quality and properties^26^. Phenomena such as drainage, imbibition and coalescence are important factors which determine the stability of liquid foams and the final solid foam structure, the latter being crucial for the mechanical performance. It is assumed that liquid drainage leads to film thinning. Films become then unstable and tend to break. The coalescence of two bubbles is triggered by the rupture of this liquid metallic film, which separates the two cells. All these phenomena have been studied in detail, especially in aqueous foams^27,28^ and also in metal foams^29,30^ on Earth.

To investigate the formation and stability of liquid metal foams, X-ray radioscopy was proven to be a powerful method^31^. To study the influence of drainage, liquid imbibition and bubble coalescence on the final solid metal foam structure, liquid metal foams have been in-situ investigated in weightlessness. For this purpose, a special experimental environment was developed and constructed with ESA support: The XRMON-MF (MF for metal foam) facility, which was successfully operated during the MASER-11 sounding rocket campaign and during the ESA PFC-46, ESA PFC-51, ESA PFC-65 and ESA PFC-67 parabolic flight campaigns (see also Table 1). Drainage, pore size, liquid fraction distribution and the number and position of film ruptures corresponding to coalescence events were identified and quantified by analysis of the X-ray images^32^.

During the ESA PFC-46, liquid drainage could be studied with and without gravity, the latter being responsible for density and pore size gradients in the final foam structure^33^. Experiments performed on MASER-11 showed that not only liquid drainage but also the gas releasing blowing agent have a major influence on bubble coalescence, and therefore on liquid foam homogeneity and stability^32,34^. A direct proof for this mechanism of coalescence is obtained by comparing samples that are foamed with the blowing agent TiH_2_ and others that owe their expansion exclusively to the gas released by the Al-Mg particles (intrinsic gas source)^35^ For this reason, we have focused in the past on investigating the stabilisation of liquid films and the development of alternative blowing agents or other foaming methods, for example with pressure manipulation^35,36^.

**Fig. 5:**
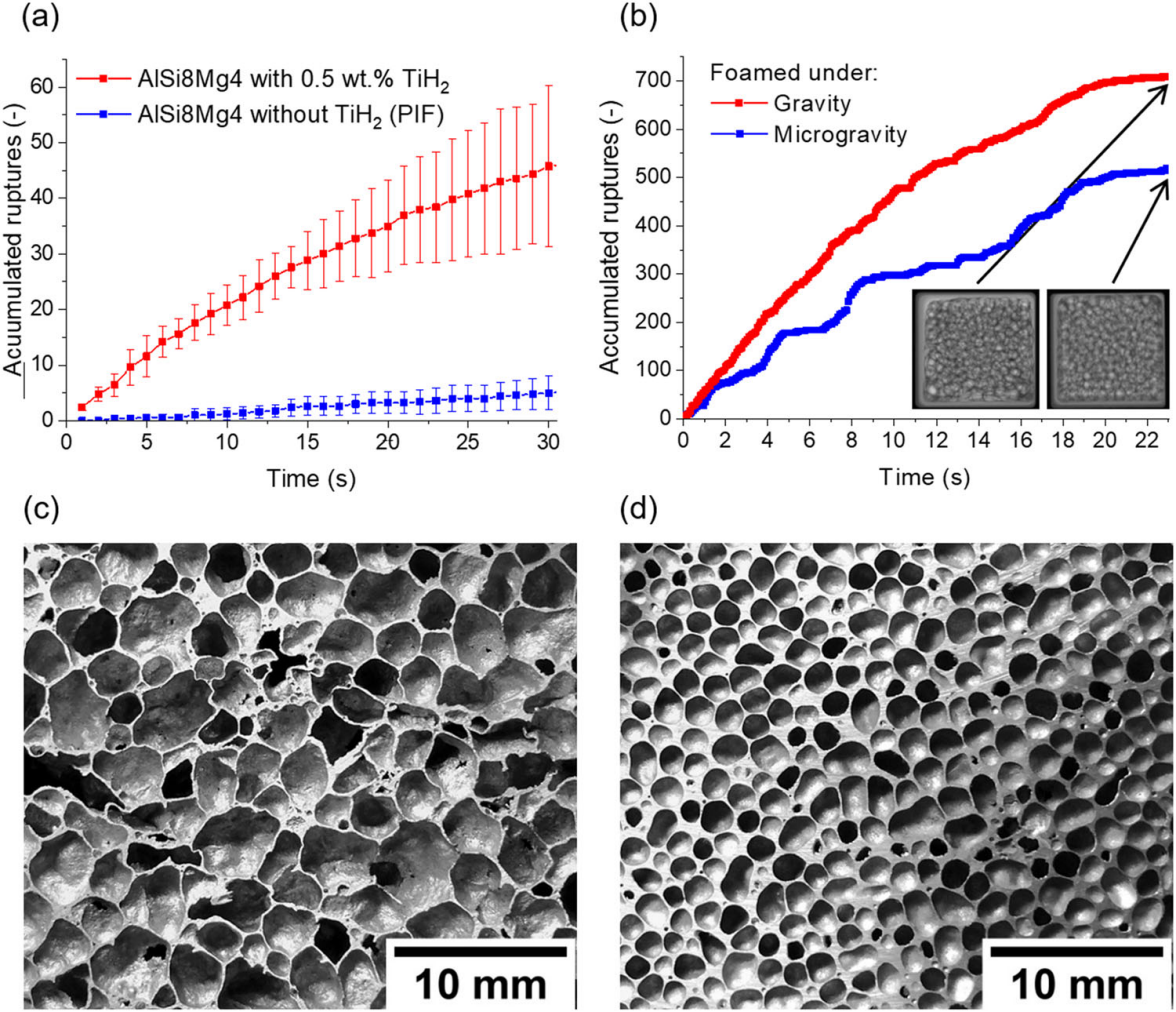
Analysis of film rupture events in AlSi8Mg4 alloy foam. **a** Accumulated number of film rupture events of the commercially available AlSi8Mg4 alloy foam produced with and without TiH_2_ as blowing agent. The ruptures were automatically calculated from the X-ray images by image analysis over a period of 30 s. The samples were of similar porosity. **b** Film rupture events in AlCu10Mg15 alloy foam determined by analysing X-ray images obtained under normal and microgravity conditions. The insets show two images of the final state after 21 s. The foam produced under microgravity conditions is clearly more homogeneous and has ~29% less film ruptures and therefore fines pores. **c** Cross-section of an AlSi8Mg4 foam blown with TiH_2_ compared with (**d**) AlCu10Mg15 foam produced without TiH_2_ featuring a more homogeneous distribution of smaller pores^37^.

Figure 5a shows a comparison of the accumulated number of rupture events for the commercial foam alloy AlSi8Mg4 (in wt.%) foamed with and without the standard blowing agent TiH_2_ by the pressure-induced foaming process^36^. The number of ruptures is about an order of magnitude higher for foams produced with TiH_2_. We have found that the blowing agent particles generate locally high gas pressures that lead to film ruptures^37^. This is not the case for foams without active gas-releasing particles.

In addition, a new foaming method has been developed that uses only gas from the surfaces of the powder particles, specially from Mg-containing powders^35^. With these samples, the bubble pressures are small, and the gas-releasing particles are evenly distributed, resulting in smaller and more homogenous distributed cells. The accumulated film ruptures and coalescence are up to ~40% higher under gravity conditions, as shown in Fig. 5b. This allowed a comparison of coalescence evolution with gravity and blowing agent-free samples of a slightly modified alloy (AlCu10Mg15). A comparison of a cross-section of the foam structure of the commercial AlSi8Mg4 alloy (Fig. 5c) with that of AlCu10Mg15 (Fig. 5d) shows that the latter has a finer and more homogeneous structure with rounder cells because of less coalescence. We draw the conclusion that drainage and coalescence are the main phenomena to be avoided to create superior solid foam structures.

During the ESA PF-51 parabolic flight campaign, the reaction of the liquid in the foam could be studied with a faster detector under changing gravity conditions (1 g → 1.8 g → 0 g → 1.8 g → 1 g, giving approximately 24 s and 22 s at 1.8 g and 0 g respectively)^38,39^. The free drainage after a microgravity phase at the onset of gravity in a liquid metal foam was characterised in-situ by quantitative analysis of the evolution of the liquid fraction distribution obtained from the X-ray radioscopy and analysed in terms of the foam drainage equation^40^. The results made it possible to obtain values for the first time of the apparent surface tension and viscosity of liquid metal foams which are necessary, for example, for simulations.

## Diffusion in melts

**Fig. 6:**
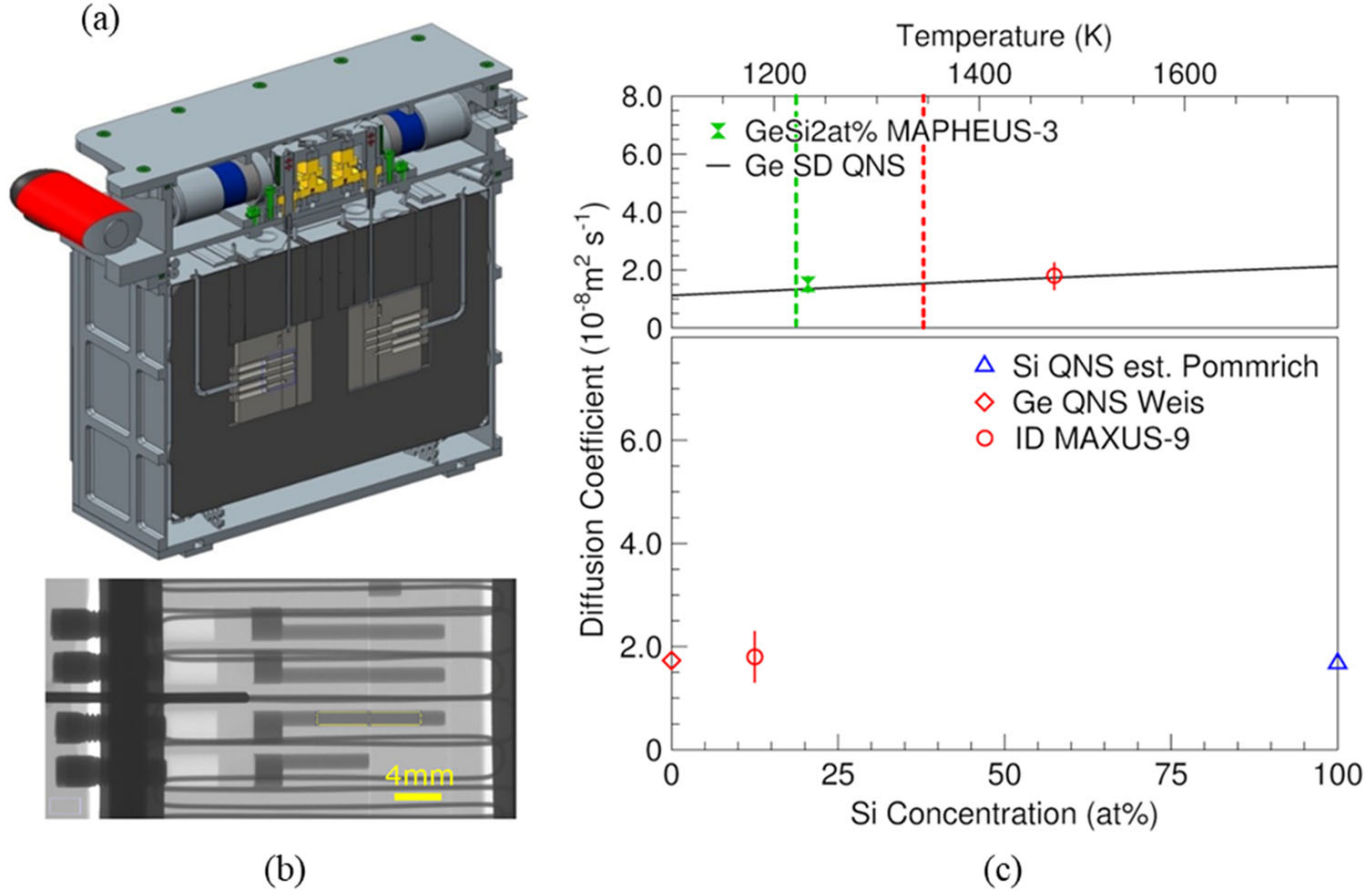
XRMON-DIF2 device and analysis of diffusion experiments. **a** XRMON-DIF2 diffusion vacuum assembly (DiVa) insert. The insert contains two ultra-high temperature shear-cell furnaces embedded in graphite foam. At the top, two stepper motors driving the respective shear-actuator (vertical grey rod in the centre) are shown. The DiVa is positioned as experimental device between X-ray source and detector with the beam transmitted perpendicular to the visible shear-cell surfaces. **b** X-radiography image of the processed shear-cell after linear shear actuation. The sample (large horizontal rods) and their reservoirs (dark grey part connected to the rods) are shown between the heater wires (thin dark lines). The dark rod inserted centrally from the left into the furnace corresponds to the thermocouples inserted into the furnace. Four different alloy compositions result in three diffusion couples after shear actuation. **c** Comparison of self- and interdiffusion experiments: Top: the solid line shows the temperature dependent self-diffusion (SD) of liquid Ge from quasi-elastic neutron scattering (QNS)^63^. Comparison with low Si-content interdiffusion experiment (Ge-GeSi2at% hourglass) from MAPHEUS-3 (microgravity) with the diffusion coefficient effectively corresponding to the Si self-diffusion. Also shown is the interdiffusion coefficient (GeSi10at%-GeSi15at% circle) obtained aboard MAXUS-9. The dashed lines (left to right) indicate the liquidus temperatures of GeSi2at% and GeSi12.5at% with GeSi15at% about 20 K higher. It has to be noted that due to non-linear corrections required for the MAXUS-9 diffusion experiments a systematic error in this data cannot fully be ruled out. Bottom: Interdiffusion coefficient obtained aboard MAXUS-9 compared with Ge and Si self-diffusion coefficients. Si self-diffusion was estimated from a quasi-elastic neutron scattering experiment by Pommrich on SiNi5at%^64^.

Self-, tracer- and chemical diffusion processes are important to understand mass transport in materials. A recent overview published by Du et al. describes the different experimental techniques and different types of diffusion for aluminium alloys^41^. In particular, for self-diffusion measurements using capillary techniques and isotopes it was shown that ground-based experiments are severely affected by buoyancy effects and that microgravity experiments are able to provide buoyancy-free conditions^42,43^. Experimental techniques were further refined by introducing shear-cells both for 1 g and microgravity experiments avoiding the impact of sedimentation and segregation during melting and freezing of the materials subject to study^44–46^. A focus was directed to chemical diffusion in liquid alloys since it is of particular relevance in structure formation and growth processes, such as they occur e.g., during solidification of metal alloys. The measurement of chemical diffusion in the previously cited experiments involves a measurement of the elemental composition along a processed – melted, annealed, and solidified – diffusion couple initially consisting of two thin rods of different composition. Using shear-cells, the two samples are separated during heating and the liquid column is segmented before cooling thereby avoiding sedimentation and segregation effects. However, even such experiments may show a large scatter in the measured data. Additional mass transport that affects the measurement was discussed by Müller and Müller-Vogt^47^. It was realised that in-situ monitoring is required to further improve the quality of the data by identifying effects that disturb the measurements. As part of XRMON it was shown that X-ray radioscopy allows in-situ monitoring of the chemical diffusion process^48^. Later on, it was shown that disturbing effects, arising from bubble formation or incomplete contacts between the samples and/or container walls, may still persist and can lead to large deviations in the diffusion coefficients from their real value^49^. Hence, further improvements were devised by not only using long-capillaries with X-ray radioscopy but to develop suitable X-ray transparent linear shear/sliding cell furnaces^50–52^. It was recently demonstrated that in-situ monitoring the diffusion process by X-ray radioscopy enables us to determine chemical and Soret-diffusion coefficients in binary metallic alloys directly^53^. For multicomponent alloys, X-ray radioscopy does not allow for a direct measurement. However, X-ray radioscopy is essential to monitor the diffusion process and rule out disturbing effects. For selected binary alloys sounding rocket (MAPHEUS) experiments using the XRISE-M facility^24^ were compared with ground-based experiments^54,55^. It was shown that diffusion experiments can be successfully carried out on ground, if a stable density-layering of the liquids is established in the sample. Therefore, a larger database of accurate diffusion coefficients in liquid binary alloys can be established at comparatively low cost. Aboard MAXUS-9, an ultra-high temperature linear-shear cell furnace – developed at DLR together with Airbus - was monitored within SSCs XRMON-DIF2 experiment facility to study chemical diffusion in binary Al-Ti alloys and in Si-Ge. Si-Ge data are shown in Fig. 6. For multicomponent alloys, multi-slice shear cell furnaces enable chemical diffusion experiments as well as combined self- and interdiffusion experiments^56^. In these experiments a stable density-layering can in most cases not be achieved. Hence, microgravity experiments remain essential. First experiments on thermo-diffusion in a binary Al-Ni alloy showed that X-ray radioscopy enables one to determine Soret-coefficients and chemical diffusion coefficients simultaneously and in-situ^51^. However, different to the before described chemical diffusion experiments which take a few to a few tens of minutes, the processing times for thermo-diffusion are typically a few hours. This will be possible in future for binary alloys using ESA’s XRF-ISS facility. Coupled with multi-slice shear-cells combined chemical and self-diffusion experiments can be carried out using X-ray radioscopy to monitor the process. To this end, new space activities using micro-launchers and new orbital platforms will be an excellent opportunity. Such platforms are currently devised for advanced materials processing and manufacturing. Coupled with inexpensive return capabilities they will make long-term experiments in reduced gravity conditions more largely available.

## Discussion

We have provided an overview of experiments carried out in the framework of the ESA XRMON project in microgravity environment, on metal alloys, with in-situ and real-time observation. Experiments were performed on board sounding rockets and during parabolic flight campaigns. It has been shown that microgravity experimentation offers a unique and efficient way to perform in-depth analyses of liquid aluminium alloy processing such as solidification, foaming or diffusion phenomena. Moreover, comparative studies of experiments on Earth and in microgravity are very helpful in providing new insights into the effects of gravity. It is worth noting that only a small number of experimental conditions have been hitherto investigated due to the limited number of opportunities. More microgravity flights will be run in the future, expanding the range of studied cases (other metallic systems such as binary Mg-based alloys and multicomponent alloys) as well as the investigation of new phenomena. Furthermore, the development of an experimental facility for the ISS, or some other space station equipped with in-situ monitoring by using X-ray radioscopy, would enable multiple experiments to be performed with the same equipment, and so give access to the study of steady-state conditions since the microgravity time on board such a platform is not limited by nature. A larger study in which experimental variables, such as alloy composition and cooling rate, could be controlled at many set levels, would be possible on the ISS and would extend our knowledge of these metallurgical processes. It would also provide a greater volume of experimental data and therefore statistics on what are essentially stochastic phenomena.

Among the possible directions for further technical developments in the long term, the improvement of the spatial resolution by using brilliant X-ray sources with smaller focal spots would open the way to using microgravity platforms for the study of metal alloys exhibiting eutectic and peritectic microstructures whose typical size is of the order or below 1 µm. In the same way, the development of compact furnaces able to reach higher temperatures would allow the investigation of alloys of industrial interest such Ni-based superalloys or steel as recently performed at synchrotron sources^57–59^.

In the case of solidification studies, the small size of the dendrite tip with respect to the thickness of the sample means that the dendrite growth kinetic can be considered close to the three-dimensional case^60^, but the thin sheet shape of the sample induces confinement effects impacting the grain development, which is a limitation to the extension of the results to bulk configurations. Carrying out post-mortem analyses such as tomography and EBSD on a systematic basis can help contravene these limitation^25,61^.

Ultimately, with the continuous progress of laboratory X-ray sources and detector efficiency, the application of X-ray tomoscopy could be envisaged in the future for direct three-dimensional analyses with relevant spatial and temporal resolutions, as already implemented at synchrotron sources^37,62^. Results from such studies would be an invaluable source of benchmark data necessary for the validation of theoretical models and numerical simulations used for industrial applications.

## Methods

### Laboratory X-ray radioscopy

**Fig. 7:**
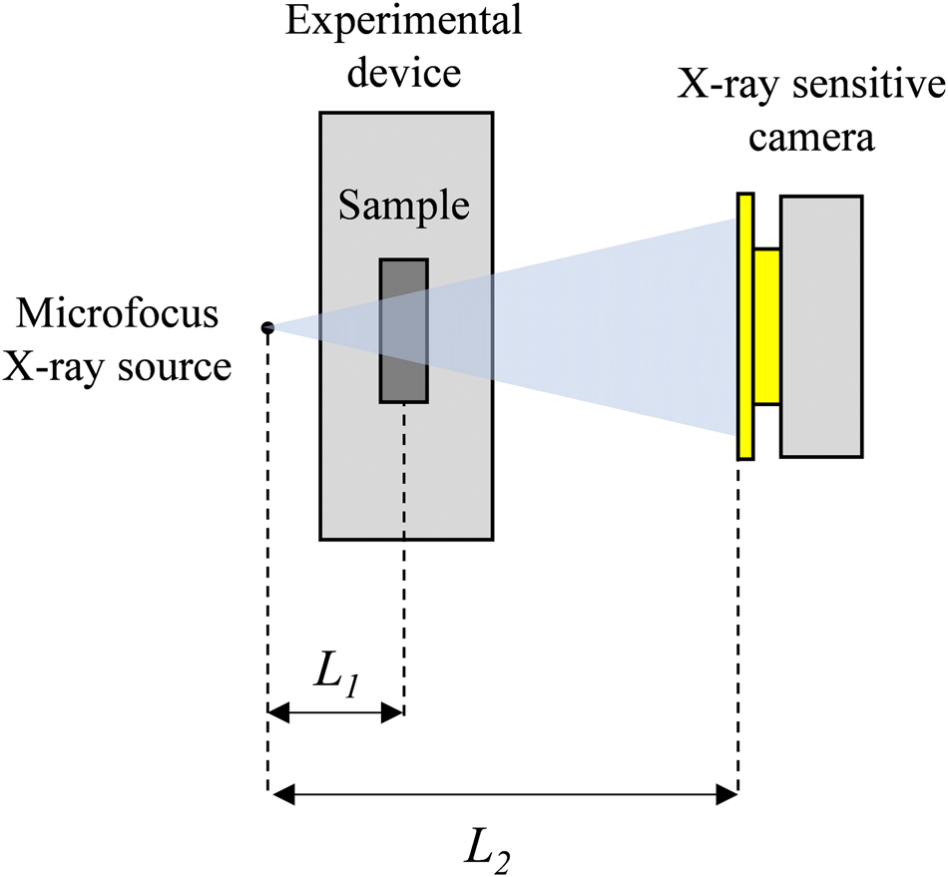
Laboratory X-ray radioscopy. Schematic illustration of the implementation of X-ray radioscopy with a laboratory micro-focus X-ray source emitting a conical beam.

In-situ observations have been carried out by using laboratory X-ray radioscopy. A general sketch of the used imaging systems is shown in Fig. 7. It comprises three parts: the X-ray source, the experimental device, and the X-ray sensitive camera. The main challenge is to find a compromise between the energy of the X-ray source, the sample thickness, and the material constituents to obtain a sufficient contrast in the images but also between the source, sample, and detector positions so that the required resolution is fulfilled, and enough photons can reach the detector.

The microfocus X-ray sources used were transmission-type X-ray tubes using Mo or W targets, with 3 to 5 microns focal spots giving a sufficient photon flux to record images with acquisition frequencies below 1 Hz. Such microfocus laboratory X-ray sources deliver a conical beam leading to a magnification $M={L}_{2}/{L}_{1}$ with ${L}_{1}$ the distance between the source and the sample and ${L}_{2}$ the distance between the source and the camera. The latter distances are mainly imposed by the geometrical constraints given by the facility accommodation to the microgravity module and the magnification was of the order of 5 to 10. The transmitted beam is collected on an X-ray sensitive camera to record a two-dimensional image corresponding to the projection through the irradiated objects. The effective pixel size of the image is given by the ratio between the camera intrinsic pixel size and the magnification $M$. The effective pixel had to be of the order of a few microns to clearly resolve the features of interest in the various reported studies. It is worth noting that the spatial resolution is limited by the source spot size and is at least twice this size.

The origin of the contrasts in the recorded images is the difference in X-ray transmission between the illuminated components. Basically, the transmission $T$ depends on the thickness $\delta$ and on the linear attenuation coefficient $\mu$ according to the Beer-Lambert law $T=\frac{{I}_{t}}{{I}_{0}}={e}^{-\delta \mu }$, where ${I}_{t}$ is the transmitted intensity and ${I}_{0}$ is the incident beam intensity. The value of the coefficient $\mu$ decreases as a function of the X-ray energy and depends on the nature of the elements present in the objects as well as their concentration and mass density.

### Reporting summary

Further information on research design is available in the Nature Research Reporting Summary linked to this article.

## Data availabity

The data that support the findings of this study are available from the corresponding author upon request.

### Supplementary information

Reporting Summary
